# Low Preoperative Platelet Count Is Associated With Deep Surgical Site Infection in Knee Arthroplasty

**DOI:** 10.1016/j.artd.2026.101989

**Published:** 2026-03-26

**Authors:** Jeffrey M. Brown, Trevor S. Lloyd, Mario Eusebio, Autreen Golzar, Alan Li, Joshua Mehany, Rene F. Chun, John S. Adams, Michael R. Yeaman, Edward J. McPherson, Nicholas M. Bernthal

**Affiliations:** a Department of Orthopaedic Surgery, David Geffen School of Medicine at UCLA, Los Angeles, CA, USA; b David Geffen School of Medicine at UCLA, Los Angeles, CA, USA; c University of California, Los Angeles, CA, USA; d Divisions of Molecular Medicine and Infectious Diseases, Department of Medicine, The Lundquist Institute for Biomedical Innovation, Harbor-UCLA Medical Center, Torrance, CA, USA; e The Lundquist Institute for Biomedical Innovation at Harbor-UCLA Medical Center, Torrance, CA, USA

**Keywords:** Knee arthroplasty, Periprosthetic infection, Risk factors, Thrombocytopenia, Platelet count

## Abstract

**Background:**

Knee arthroplasty (KA) significantly improves pain and function in gonarthrosis. Despite its success, major complications like deep surgical site infection (SSI) remain a concern. Infection treatments often require aggressive protocols, including implant exchange and prolonged antimicrobial therapy, emphasizing the need for preoperative patient optimization to mitigate infection risk. Preoperative platelet count has been proposed as a predictive marker for postoperative infection. Beyond their hemostatic role, platelets possess antimicrobial properties and are considered the first line of defense against microbial proliferation within the surgical site. This database review aims to assess the relationship between preoperative platelet count and deep SSI.

**Methods:**

The National Surgery Quality Improvement Program was queried for patients who underwent primary total or unicompartmental KA between 2015 and 2022 and developed deep SSI within 30 days. Postoperative outcomes were analyzed with respect to platelet count as a continuous and categorical variable.

**Results:**

Deep SSI was significantly more common in patients with preoperative platelet counts <150,000/*μ*l compared to those with higher counts (0.61% vs 0.30%; *P* < .001). Regression analysis assessed the independent association of preoperative platelet count with deep SSI. The odds ratio for deep SSI with platelet count <50k/*μ*l was 2.47 (1.42-4.28; *P* = .001), 1.87 (1.08-3.24; *P* = .026) for platelet count between 50-100k/*μ*l, and 1.62 (1.32-2.00; *P* < .001) for platelet count between 100-150k/*μ*l, all in reference to patients with platelet counts >150k/*μ*l.

**Conclusions:**

Thrombocytopenia (<150,000 platelets/*μ*l) is significantly associated with a higher incidence of deep SSI following KA. Preoperative platelet count may serve as a useful marker for infection risk.

## Introduction

Knee arthroplasty (KA), including unicompartmental KA and total KA (TKA), provides significant pain relief and improved function in patients suffering from end-stage gonarthrosis [1,2]. KA is a reliably successful surgery, with an estimated 480,000 TKAs performed annually in the United States as of 2019, and projected increases ranging from 790,000 to 935,000 by 2030 [3,4]. In the years 1991 through 2010, the incidence of TKA doubled per capita from 31 to 62 per 10,000 Medicare enrollees annually [5]. Given the historical and projected rise in demand for KA, optimizing preoperative health remains critical to minimize adverse perioperative events.

Despite ongoing advancements in surgical techniques and postoperative care, KA is not without risk. Among the potential complications, periprosthetic joint infection (PJI) is particularly noteworthy due to its significant impact on patient outcomes and healthcare resources [6]. Clinically, PJI is characterized by robust bacterial adherence to implants in the form of a biofilm, conferring increased resistance to antimicrobial therapy [7,8]. Effective treatment of PJI typically requires aggressive surgical intervention, such as an exchange protocol, combined with prolonged antimicrobial therapy. These protocols, while essential, impart patient morbidity and even mortality [9,10].

PJI also poses a substantial economic burden. The incidence of PJI following primary KA is estimated at 1-2%, encompassing infections that can arise within a few weeks to several years postoperatively [7,11,12]. Although studies evaluating early postoperative infections over shorter periods often report lower incidence rates, the cumulative burden of PJI remains considerable [13,14]. In the United States, annual direct hospital costs for treating hip and knee PJI are projected to exceed $1.85 billion by 2030 [4]. Effective infection prevention strategies are crucial for improving patient outcomes and alleviating the growing economic strain.

Risk factors for PJI are well described, and risk scoring of patients with PJI can delineate high- to low-risk categories according to host and limb health [[15], [16], [17]]. However, despite these insights and mitigation efforts, the incidence of PJI has remained consistent over time, suggesting its prevalence will rise in proportion to the increasing incidence of KA procedures [18].

Despite substantial progress in identifying risk factors for PJI, there remains a need to elucidate host-specific factors contributing to infection risk. Several studies have described the role of platelets in the host immune response and antimicrobial defense beyond their classically understood role in hemostasis [[19], [20], [21]]. It is hypothesized that platelets serve as the first line of defense against an organizing deep infection [21]. Thrombocytopenia (<150,000 platelets/*μ*l) compromises this defense, leading to an increased risk of infection [22]. Although discerning the impact of thrombocytopenia from other comorbidities affecting platelet quantity and function is challenging, causal associations between thrombocytopenia and orthopedic infections have been demonstrated [[23], [24], [25]]. This study aims to explore the relationship between preoperative platelet count and the frequency of deep surgical site infection (SSI) following KA. We hypothesize that reduced serum platelet count is associated with an increased rate of deep infection.

## Material and methods

The American College of Surgeons National Surgery Quality Improvement Program (NSQIP) is an administrative database that documents preoperative and postoperative clinical data and tracks outcomes within 30 days of surgery with the intent to identify preventable complications. The NSQIP database was used to identify 493,614 patients who underwent primary unicompartmental or total knee arthroplasty between years 2015 and 2022 (current procedural terminology codes 27446 and 27447, respectively). There were 32,088 patients excluded for having no reported preoperative platelet count, leaving 461,526 patients for analysis. KA patients were grouped into the following subgroups: <150,000, 150-200k, 200-250k, 250-300k, and >300k platelets/*μ*l. The threshold of <150,000 platelets/*μ*l was used to define thrombocytopenia [26].

Patient age and sex were reported, as well as select comorbidities and potential confounding factors including smoking status, corticosteroid use, obesity, and diabetes. The American Society of Anesthesiologists (ASA) preoperative health score was also assessed for each patient. Complications within 30 days, including deep SSI, wound dehiscence, and revision surgery related to the primary procedure were documented using the NSQIP database. Deep SSI was a composite variable of deep incisional SSI and organ space SSI, as defined in the NSQIP database. Of note, NSQIP classifies infections that originate as organ space SSI and drain through the incision as deep incisional SSI. This necessitated our use of the composite variable designated as “deep SSI” [[27], [28], [29]]. The deep SSI composite variable includes but does not exclusively constitute occurrences of PJI in this cohort but rather was used as a surrogate variable for deep postoperative infections in KA patients. Wound dehiscence—a standard variable tracked in NSQIP and defined as the reopening of a surgical wound along the incision line—was documented as a separate complication. Revision surgery related to the primary procedure was identified by tracking any unplanned return to the operating room within the 30-day postoperative period, including surgical interventions required to address complications such as infection or mechanical failure. Of patients who returned to the operating room within 30 days for a complication related to the primary procedure, infectious causes for revision surgery were identified with standardized International Classification of Diseases (ICD-10) T84.5 family codes.

Preoperative platelet count was analyzed as a continuous variable using independent sample *t*-tests and Analysis of Variance (ANOVA) tests, and as a categorical variable using chi-square tests with subgroup analysis. *P* values <0.05 with a confidence interval of 95% were considered statistically significant. Additionally, a binomial logistic regression was performed to assess the influence of the previously mentioned variables on deep SSI. Statistical analysis was performed with IBM SPSS Statistics version 29.0.1.0, and figures were produced with GraphPad Prism version 9.5.1.

## Results

**Figure 1:**
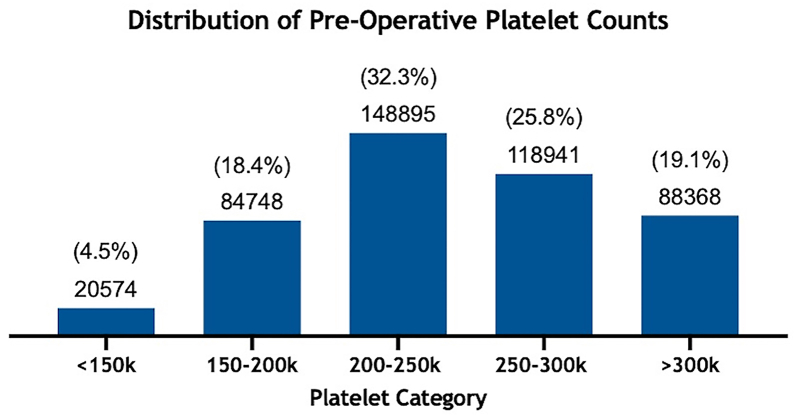
Histogram showing distribution of 461,526 patients after dividing the study population into 5 groups based on their preoperative platelet count.

Across 461,526 KA patients with a documented preoperative platelet count, 20,574 (4.5%) patients had a platelet count <150k/*μ*l, 84,748 (18.4%) patients were between 150-200k/*μ*l, 148,895 (32.3%) patients were between 200-250k/*μ*l, 118,941 (25.8%) patients were between 250-300k/*μ*l, and 88,368 (19.1%) patients had a platelet count >300k/*μ*l (Fig. 1).

Table 1 reviews patient demographics, comorbidities, and complications within this cohort. Of the KA patients, 279,931 (60.7%) were male, and 181,578 (39.3%) were female. There were 95,813 (20.8%) patients under age 60, 195,883 (42.4%) patients between ages 60 and 70, and 169,809 (36.8%) patients over age 70. There were 84,255 patients (18.3%) with diabetes mellitus (non-insulin or insulin dependent), 35,285 patients (7.6%) with a current or recent smoking history, and 17,126 (3.7%) patients reporting daily or recent corticosteroid use. Across body mass index (BMI) categories, 9.5% of patients had a BMI <25, 26.7% had a BMI between 25 and 30, 29.8% had a BMI between 30 and 35, and 34.1% of patients had a BMI over 35. 48.6% of the cohort had an ASA score of 1 or 2 (healthy or mild systemic disease), and 51.4% of patients had an ASA score of 3-5, denoting severe systemic disease or worse.

**Table 1:** Baseline patient characteristics.

Characteristic	n (%)
All patients (with defined platelet count)	461,526 (100)
Sex	
Male	279,931 (60.7)
Female	181,578 (39.3)
Age	
<60	95,813 (20.8)
60-70	195,883 (42.4)
>70	169,809 (36.8)
Diabetes	
No	377,251 (81.7)
Yes	84,255 (18.3)
Smoking	
No	426,220 (92.4)
Yes	35,285 (7.6)
Steroids	
No	444,381 (96.3)
Yes	17,126 (3.7)
Deep infection	
No	460,105 (99.7)
Yes	1421 (0.3)
Dehiscence	
No	460,450 (99.8)
Yes	1052 (0.2)
Return to operating room within 30 days (related to primary procedure)	
No	457,565 (99.1)
Yes	3920 (0.9)
Body mass index	
<25	43,565 (9.5)
25-30	122,779 (26.7)
30-35	137,002 (29.8)
35-40	92,861 (20.2)
>40	64,095 (13.9)
ASA status	
Class 1-2	224,138 (48.6)
Class 3-5	236,824 (51.4)

ASA, American Society of Anesthesiologists Physical Status Classification System.

The incidence of deep SSI within 30 days in this cohort was 1421 (0.3%), which included deep incisional and organ space SSI as described in the NSQIP database. The average platelet count for patients with deep SSI was significantly lower than patients with no infectious complications when assessed as a continuous variable (243.52k vs 247.62k, respectively, *P* = .009).

The incidence of wound dehiscence was 1052 (0.2%), with no significant differences in mean platelet count between patients with and without wound dehiscence (247.61k vs 248.66k; *P* = .884).

**Figure 2:**
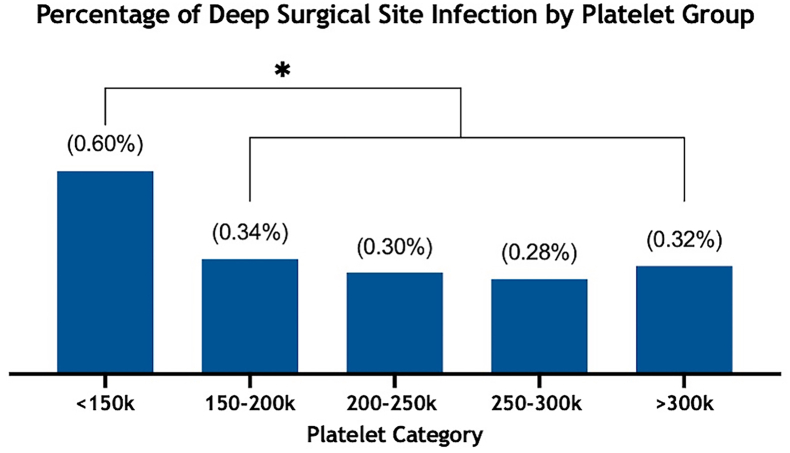
Histogram showing the percentage of the study population with deep surgical site infection after dividing the population into 5 groups based on their preoperative platelet count. ∗*P* < .001.

The association between preoperative platelet count and infection rate was also analyzed with platelet count as a categorical variable (Fig. 2). The incidence of deep SSI for patients <150k was 124 (0.60%), 291 (0.34%) at 150-200k, 386 (0.30%) at 200-250k, 335 (0.28%) at 250-300k, and 285 (0.32%) at >300k (Fig. 2). The <150k platelet subgroup had significantly higher frequency of deep SSI compared to the higher platelet subgroups (*P* < .001) (Fig. 2).

As an additional method of evaluating the frequency of infection in association with preoperative platelet count, patients who underwent revision surgery due to an infectious complication within 30 days of their primary KA were compared to patients who did not undergo revision surgery. There were 3920 (0.9%) instances of revision surgery within 30 days, 696 (0.15%) of which were due to infectious causes. This is a subset of the 1421 cases (0.3%) of deep SSI reported in the cohort, as not all infections resulted in revision surgery. The frequency of revision surgery due to infectious complications was likewise higher in patients with preoperative platelet counts <150k/*μ*l (24.6%) compared to patients with preoperative platelet counts >150k/*μ*l (19.5%; *P* < .001).

Additionally, several demographic and clinical features reported in this study have known independent influence on both platelet counts and infection risk. To control for the potential confounding effects of these variables on observed infection rates and platelet count, multiple subanalyses were conducted.

A cohort of nonsmoking, nondiabetic patients who did not use corticosteroids were assessed (n = 335,136). Even when accounting for these variables, within this cohort, patients were still significantly more likely to have deep infection with a platelet count <150k/*μ*l (0.51% vs 0.25%; *P* < .001).

This relationship between thrombocytopenia and deep surgical infection persisted when separating patients into cohorts of comparable age. Within each age range, patients with platelet counts <150k/*μ*l showed consistently higher incidence of deep infection (0.89% vs 0.38% in patients <60 years old, *P* < .001;_0.66% vs 0.27% in patients 60-70, *P* < .001; 0.49% vs 0.27% in patients older than 70 years old, *P* = .003). The same finding occurred when looking into a cohort consisting of only males (0.72% vs 0.41%; *P* < .001) or only females (0.41% vs x 0.22%; *P* < .001).

In a cohort of patients without wound dehiscence, patients with preoperative thrombocytopenia (<150k platelets/*μ*l) had a higher incidence of deep SSI (0.55% vs 0.26%; *P* < .001). This difference did not persist when analyzing the cohort of patients (n = 1052) with wound dehiscence (18.46% vs 18.33%; *P* = .310).

In a cohort of 173,816 patients with ASA class 1-2 and BMI<35, patients with preoperative platelet counts <150k still had significantly higher rates of infection (0.43% vs 0.19%; *P* = .002).

In an analysis of ASA class 1-2, BMI <35, nonsmoking, nondiabetic patients with no wound dehiscence or corticosteroid use (n = 143,819), patients with platelet counts <150k again showed higher incidence of infection (0.30% vs 0.15%), though this difference did not reach statistical significance (*P* = .051).

As a final method of statistical analysis, a binomial logistic regression was performed to assess the independent association of sex, age group, diabetes, smoking status, corticosteroid use, preoperative platelet count, BMI, and ASA status on deep SSI. Platelet count was further subdivided into groups of <50k, 50-100k, 100-150k, and >150k to more finely assess the impact of thrombocytopenia. This regression analysis demonstrated that thrombocytopenia was indeed independently associated with an increased risk for deep SSI, with increased risk in parallel with increased severity of thrombocytopenia. The odds ratio for deep SSI with platelet count <50k was 2.47 (1.42-4.28; *P* = .001), 1.87 (1.08-3.24; *P* = .026) for platelet count between 50-100k, and 1.62 (1.32-2.00; *P* < .001) for platelet count between 100-150k, all in reference to patients with platelet counts >150k (Table 2). Other variables significantly increasing the risk for deep SSI included male sex, smoking, corticosteroid use, high ASA class, and morbid obesity (BMI >35). The full results of the logistic regression are available in Table 2.

**Table 2:** Multivariable binomial logistic regression analysis of risk factors for deep SSI.

Characteristic	Odds ratio (CI)	*P* value
Sex		
Female (reference)		
Male	1.98 (1.78-2.22)	<.001
Age		
<60 (reference)		
60-70	0.83 (0.72-0.95)	.005
>70	0.92 (0.79-1.06)	.244
Diabetes		
No (reference)		
Yes	1.08 (0.948-1.23)	.251
Smoking		
No (reference)		
Yes	1.97 (1.69-2.29)	<.001
Corticosteroids		
No (reference)		
Yes	1.58 (1.26-1.98)	<.001
Platelet category		
<50k	2.47 (1.42-4.28)	.001
50-100k	1.87 (1.08-3.24)	.026
100-150k	1.62 (1.32-2.00)	<.001
>150k (reference)		
Body mass index		
<25 (reference)		
25-30	0.87 (0.69-1.09)	.226
30-35	1.07 (0.86-1.33)	.556
35-40	1.29 (1.02-1.61)	.031
>40	1.92 (1.52-2.42)	<.001
ASA status		
Class 1-2 (reference)		
Class 3-5	1.42 (1.26-1.59)	<.001

ASA, American Society of Anesthesiologists Physical Status Classification System.

## Discussion

The growing frequency of KA as a commonplace orthopaedic procedure in the aging US population reinforces the importance of identifying and optimizing risk factors for postoperative complications, including deep SSIs such as PJI. A comprehensive understanding of all potential risk factors is critical for the development of effective mitigation strategies. Several risk factors for deep SSI and PJI are well described, including advanced age, sex, uncontrolled diabetes, smoking, poor wound healing, and states of immunosuppression, and these factors also influence platelet count. This study proposes that preoperative platelet count is associated with the risk of deep SSI following KA procedures. Our analysis revealed a correlation between preoperative thrombocytopenia and deep SSI within 30 days of KA, even when controlling for comorbidities and demographic factors influencing platelet count.

This article highlights a potential area of further study in which platelets play a vital role in the immune system by mechanistically restricting the initial proliferation of microbes, thereby giving other immune cells the time necessary to initiate a robust counter response. The mechanisms by which platelets limit initial microbial growth within the synovial joint are complex and distinct from their traditional role in thrombosis [30,31]. These antimicrobial properties include pathogen recognition, generation of reactive oxygen species, release of microbicidal proteins, and recruitment and activation of immune cells [20,32,33]. A deficiency in the level of serum platelets may represent an independent risk factor for deep infection, similar to deficiencies in other immune cell lines. This study was conducted to determine if a reduced platelet level is associated with an increased rate of deep infection in KA and, if so, what platelet level places patients at an increased risk for deep infection.

Patients with preoperative platelet counts <150,000 platelets/μl were found to have a higher rate of deep infection following KA within the first 30 days of the index procedure. The selection of a 30-day period for observing deep infection rates in KA is based on the standardized follow-up period used by the American College of Surgeons NSQIP database, to provide a consistent and comparable measure of early postoperative complications, including infections. Furthermore, even after accounting for comorbidities including diabetes, current or recent smoking, and corticosteroid use, a platelet count <150k remained significantly associated with a deep infection in KA. When reviewing the demographic differences of age and sex upon mean platelet count, deep infection was still associated with a platelet count <150k, including in patients with a low ASA score (ASA 1-2). Finally, the logistic regression likewise demonstrated an independent association with preoperative thrombocytopenia and increased risk for deep SSI in a concentration-dependent manner.

It is worth drawing attention to the issue of wound dehiscence and its role as an avenue for microbial penetration. As expected, the reported frequency of deep SSI was higher in patients with knee wound dehiscence [[34], [35], [36]]. Interestingly, thrombocytopenia of <150k platelets/*μ*l was correlated with deep infection in the absence of a superficial wound dehiscence (*P* = .026). This suggests that significantly lower platelet count in patients with deep SSI is not solely due to impaired healing and wound dehiscence; it supports the notion of platelets exhibiting antibacterial properties beyond their function in hemostasis and wound healing.

This study is not the first to explore an association between platelet count and postsurgical complications, including musculoskeletal infection. In a preclinical mouse model, Greig et al. demonstrated that induced thrombocytopenia was a modifiable risk factor for periprosthetic infection in vivo and for bacterial growth ex vivo [25]. Malpani et al. reported a significant association of abnormally high or low platelet counts with major adverse events following elective primary TKA, including a significantly greater frequency of wound infection in thrombocytopenic patients [37]. While their study used a slightly lower cutoff for thrombocytopenia (≤116k) than this present study, the results are consistent in correlating this variable with elevated postoperative risk. Conversely, Kim et al. reported complications following total shoulder arthroplasty and did not observe significant differences in the frequency of infection across groups with varying degrees of thrombocytopenia [38]. These findings suggest that while thrombocytopenic patients with a platelet count below 150,000 may benefit from further optimization or closer monitoring to mitigate infection risk, this is not yet an actionable recommendation for patients with otherwise normal platelet counts. Instead, this association highlights a potential risk factor that should be discussed with patients as part of their overall perioperative risk assessment, pending further longitudinal studies to establish causality and define modifiable interventions.

It is necessary to emphasize that both thrombocytopenia and deep SSI are complex phenomena with myriad influencing variables. A direct correlation of platelet count levels to postoperative infection must be interpreted with restraint. In that light, this study has several limitations. The NSQIP database is structured to track a broad range of procedure-related complications across various surgical specialties. Thus, there are generalized infection categories (such as deep incisional and organ space SSI) which may not be clinically familiar terms commonly used in the field of orthopaedic surgery. Our composite variable (“deep SSI”) in this study overlaps with, but does not necessarily perfectly correlate or confirm, PJI. The NSQIP database also only tracks complications within 30 days of surgery, resulting in a significant underestimation of the frequency of deep SSI in this cohort. It is known that a deep infection or PJI may manifest up to 2 years from the index KA procedure, and a significant number of infectious complications are not captured in this analysis due to database constraints [39]. This 30-day reporting window is also likely responsible for the low reoperation rate in our deep SSI cohort, as it is reasonable to assume that not all patients with diagnosed infection within 30 days also underwent reoperation within 30 days. Another significant limitation includes restricted ability to assess for confounding surgical site factors such as hematoma [40,41]. Furthermore, findings from any large database must be interpreted with caution as there are invariably discrepancies in data entry from institution to institution. Despite these limitations, the observed association between thrombocytopenia and deep SSI supports our hypothesis that platelets may be an independent factor for deep infection in KA. Direct longitudinal study of these reported observations is our next step to validate our hypothesis.

## Conclusions

This study describes the correlation of preoperative thrombocytopenia with deep infection occurring within 30 days of primary KA surgery. A platelet count <150,000 platelets/μ l was associated with an increased risk of deep infection. The association between deep infection and thrombocytopenia was observed even when controlling for comorbidities and demographic factors influencing platelet count, with progressive risk for infection with increasing severity of thrombocytopenia, as demonstrated in regression analysis. Further direct longitudinal study of preoperative thrombocytopenia and PJI is required to validate preoperative thrombocytopenia as an independent risk factor for PJI.

## CRediT authorship contribution statement

**Jeffrey M. Brown:** Writing – review & editing, Writing – original draft, Visualization, Validation, Methodology, Investigation, Formal analysis, Data curation, Conceptualization. **Trevor S. Lloyd:** Writing – review & editing, Writing – original draft, Methodology, Investigation, Formal analysis, Data curation, Conceptualization. **Mario Eusebio:** Writing – review & editing, Writing – original draft. **Autreen Golzar:** Writing – review & editing, Writing – original draft, Methodology, Formal analysis, Data curation. **Alan Li:** Writing – review & editing, Writing – original draft, Visualization, Formal analysis, Data curation, Conceptualization. **Joshua Mehany:** Writing – review & editing, Writing – original draft, Conceptualization. **Rene F. Chun:** Writing – review & editing, Writing – original draft, Visualization, Formal analysis, Conceptualization. **John S. Adams:** Writing – review & editing, Writing – original draft, Supervision, Project administration, Conceptualization. **Michael R. Yeaman:** Writing – review & editing, Writing – original draft, Supervision, Methodology, Conceptualization. **Edward J. McPherson:** Writing – review & editing, Writing – original draft, Supervision, Methodology, Conceptualization. **Nicholas M. Bernthal:** Writing – review & editing, Writing – original draft, Supervision, Project administration, Methodology, Conceptualization.

## Conflicts of interest

The authors declare there are no conflicts of interest.

For full disclosure statements refer to https://doi.org/10.1016/j.artd.2026.101989.

## Appendix A Supplementary data

Conflict of Interest Statement for Adams

Conflict of Interest Statement for Mehany

Conflict of Interest Statement for Yeaman

Conflict of Interest Statement for Bernthal

Conflict of Interest Statement for Brown

Conflict of Interest Statement for Chun

Conflict of Interest Statement for Eusebio

Conflict of Interest Statement for Golzar

Conflict of Interest Statement for Li

Conflict of Interest Statement for Lloyd

Conflict of Interest Statement for McPherson
